# Supplementary material for: Deformable image registration based on single or multi-atlas methods for automatic muscle segmentation and the generation of augmented imaging datasets
Source: PLoS One. 2023 Mar 10;18(3):e0273446. doi: 10.1371/journal.pone.0273446 (PMC10004495; doi:10.1371/journal.pone.0273446)
Supplement: S1 File — Reference muscle volumes for the 23 muscles segmented in this study. Mean and standard deviation are reported. (PDF) [file pone.0273446.s001.pdf]

Body segment	Muscle	Volume (mm³)					
		Subject 1	Subject 2	Subject 3	Subject 4	Subject 5	Mean ± Standard deviation
Hips	Adductor brevis	67057	65258	57964	58377	54244	60580 ± 4810
	Adductor longus	63097	59674	78014	66235	91674	71739 ± 11725
	Adductor magnus	456876	378554	337569	323952	281549	355700 ± 59319
	Gluteus maximus	606071	653688	474065	405632	593184	546528 ± 91987
	Iliacus	81848	113228	101089	127435	124052	109530 ± 16630
	Tensor fasciae latae	50466	49527	17359	36104	57855	42262 ± 14292
Thigh	Biceps femoris caput brevis	60861	73188	31466	80666	68629	62962 ± 17009
	Biceps femoris caput longum	127749	121698	108420	119117	95255	114448 ± 11454
	Gracilis	41912	51212	44069	47798	37633	44525 ± 4690
	Rectus femoris	117610	113081	93965	121547	125296	114300 ± 10949
	Sartorius	62725	105340	64017	63115	101729	79385 ± 19755
	Semimembranosus	122046	126232	103785	153686	98869	120924 ± 19406
	Semitendinosus	91783	95741	94701	101293	88536	94411 ± 4258
	Vastus intermedius	213619	313517	255788	277240	229770	257987 ± 35281
	Vastus lateralis	317807	351828	303185	326823	328335	325596 ± 15875
	Vastus medialis	167408	276617	223911	225695	178173	214361 ± 39012
Calf	Gastrocnemius lateralis	87151	80340	78233	86873	81386	82797 ± 3589
	Gastrocnemius medialis	141089	175820	162956	163094	122901	153172 ± 18812
	Peroneus brevis	40078	41577	33732	38826	40896	39022 ± 2799
	Peroneus longus	34349	58957	33972	25693	35069	37608 ± 11208
	Soleus	306017	405911	323367	304719	328053	333613 ± 37308
	Tibialis anterior	82749	94150	78794	74447	88936	83815 ± 7031
	Tibialis posterior	65793	90619	88953	56327	72720	74882 ± 13246

Table 1: Reference muscle volumes for the 23 muscles segmented in this study. Mean and standard deviation are reported.
