# Supplementary material for: Deformable image registration based on single or multi-atlas methods for automatic muscle segmentation and the generation of augmented imaging datasets
Source: PLoS One. 2023 Mar 10;18(3):e0273446. doi: 10.1371/journal.pone.0273446 (PMC10004495; doi:10.1371/journal.pone.0273446)
Supplement: S2 File — Registration protocol and results for the left to right (intra-subject) analysis, providing the optimal values for the nodal spacing and smoothing coefficient, two registration parameters. (PDF) [file pone.0273446.s002.pdf]

Methods

The optimal values for the two user-imposed registration parameters: the nodal spacing (NS) and smoothing coefficient were verified through a sensitivity analysis. The analysis designed for the sensitivity analysis was a simplified version of the inter-subject analysis outlined in the study. One subject was selected at random and the left limb (after reflection) of this subject was registered to the right limb (Fig. 1). The three error metrics defined in the paper (relative volume error (RVE), Dice similarity coefficient (DSC), and Hausdorff distance (HD)), were calculated for the resulting segmentations of the 23 muscles considered. 8 values were tested for the NS ([5,10,…,40] mm), and 5 values for the smoothing coefficient were tested ([1.35, 13.5, 135, 1350, 13500]). The optimal smoothing coefficient is automatically calculated within the Sheffield Image Registration Toolkit [1] (135 for this registration task) and operates on a logarithmic scale, hence the choice of two orders of magnitude greater and smaller than the automatically calculated value.

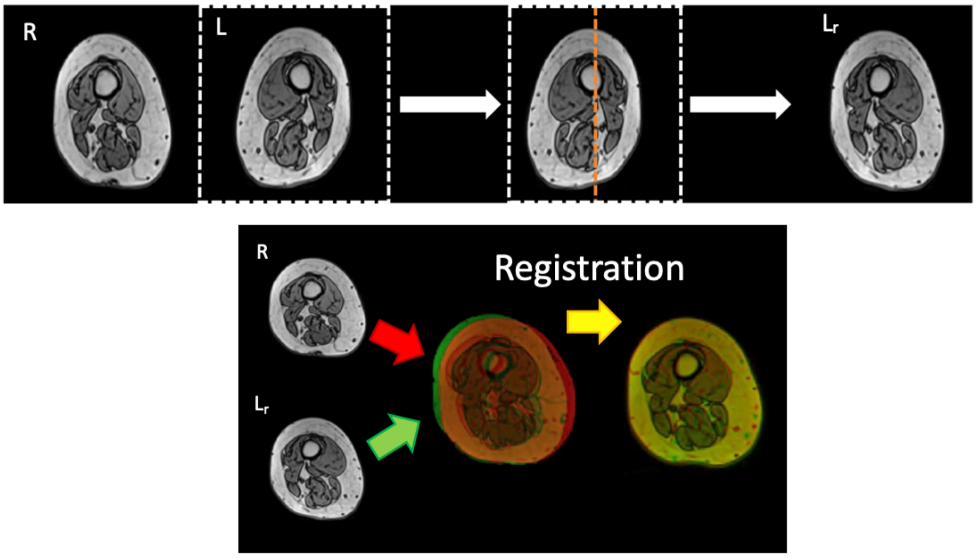

<u>Fig. S1</u>:
Top: separation of the anatomical right (R) and left (L) limb followed by a reflection of the left limb in the sagittal axis (L_r), shown for one example 2D slice of imaging data. Bottom: Registration of MR imaging data. The reflected left image (L_r) is inputted into ShIRT as the moving image (shown in green) and registered to the right image (R), inputted as the fixed image (shown in red). The registered image (right most image) shows these images after registration.

Results

The sensitivity analysis for the NS shows that the optimal nodal spacing for this segmentation task is 5 mm (Fig. 2). The RVE found presented the smallest variance, but all achieved an average RVE of close to 0%. The DSC found when using a NS of 5 mm presented the best segmentation accuracy. The HD was consistent across all NS considered.

The sensitivity analysis for the smoothing coefficient verifies that the automatically calculated smoothing coefficient was indeed the optimal value for this registration task. The RVE found for the optimal smoothing coefficient (135) presented the smallest error (Fig. 3). The DSC found when using the optimal smoothing far exceeded the other values assessed. Additionally, the HD was lower when the registration was performed using the optimal smoothing coefficient.

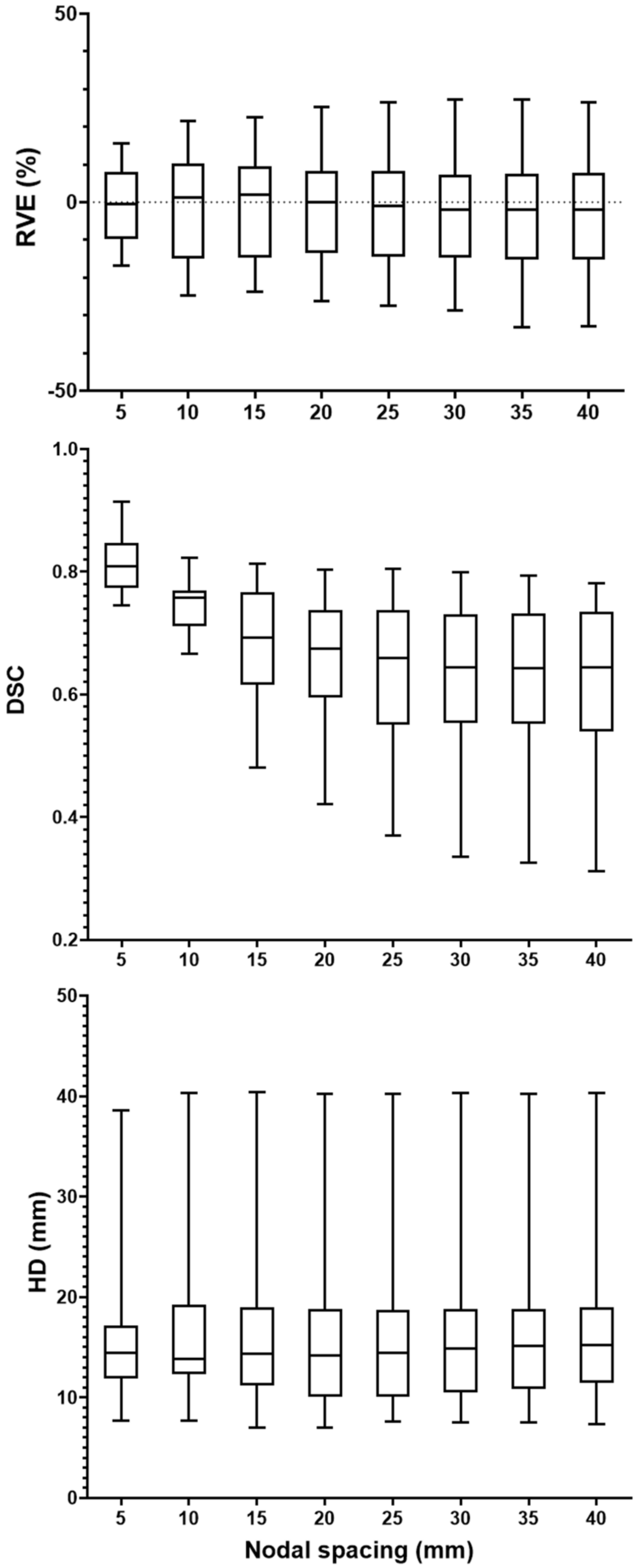

<u>Fig S2</u>:

The sensitivity analysis of the nodal spacing, comparing segmentation accuracy when using 8 values of nodal spacing with the three error metrics: RVE, DSC and HD.

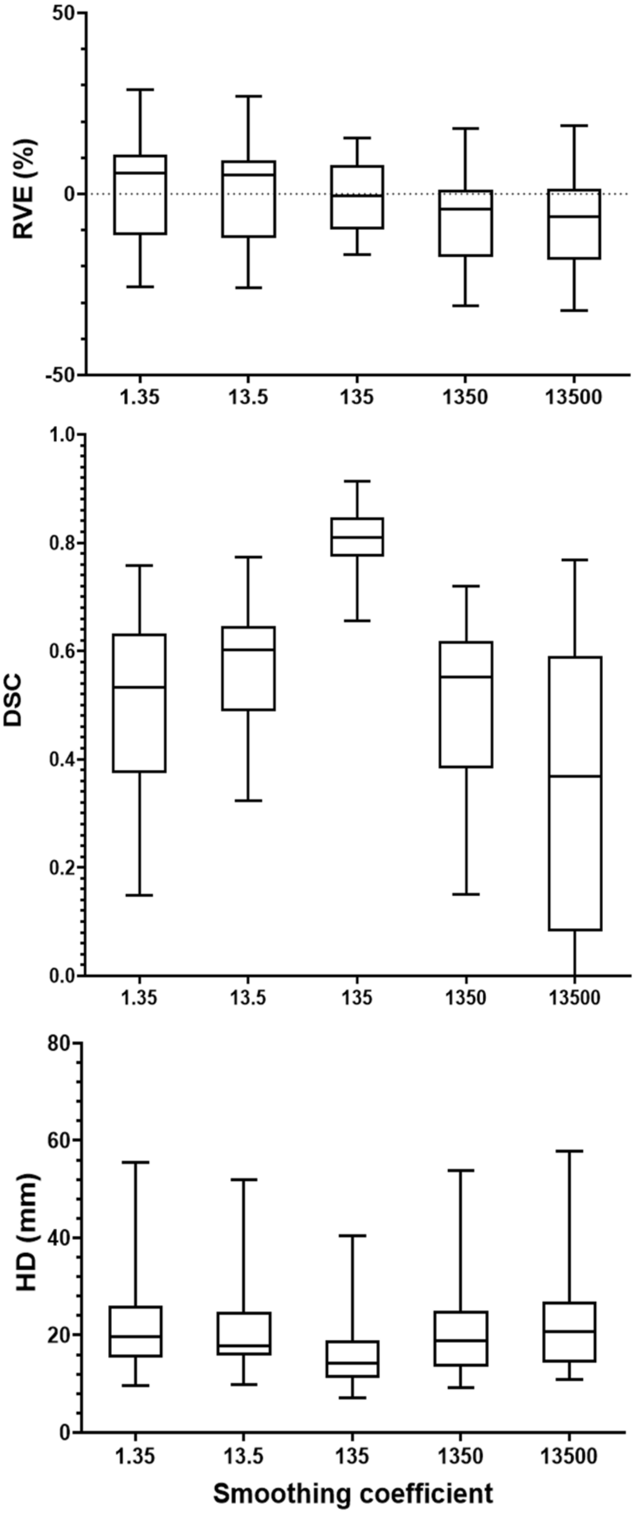

<u>**Fig S3**</u>:

The sensitivity analysis of the smoothing coefficient, comparing segmentation accuracy when using 5 values of the smoothing coefficient with the three error metrics: RVE, DSC and HD. The median value used of 135 was automatically calculated within the Sheffield Image Registration Toolkit.
