# Supplementary material for: Deformable image registration based on single or multi-atlas methods for automatic muscle segmentation and the generation of augmented imaging datasets
Source: PLoS One. 2023 Mar 10;18(3):e0273446. doi: 10.1371/journal.pone.0273446 (PMC10004495; doi:10.1371/journal.pone.0273446)
Supplement: S3 File — Display of augmented datasets for one target subject. The image on the left shows a cross- section of the target subject (Subject1) with the manual segmentations for that image shown in green. The 10 images on the right are cross-sections of the augmented datasets, generated when keeping subject 1 as the target for the registration, whilst using the other 10 subjects as the reference dataset. Segmentations are reported in blue. One augmented dataset marked with a red square did not pass the inclusion criteria, due to the discontinuity in the boundary of the body. (PDF) [file pone.0273446.s003.pdf]

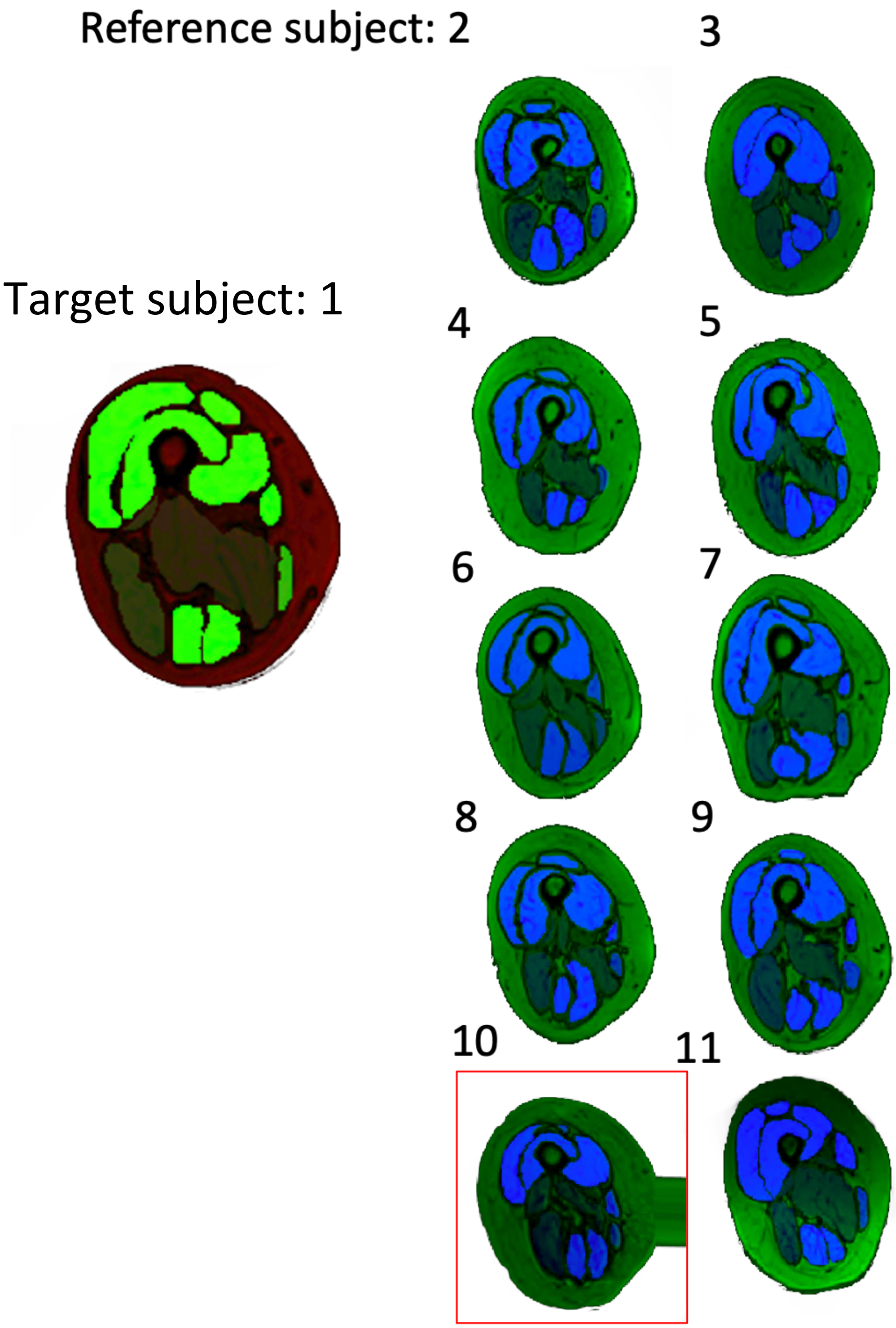

Fig. 1:
Display of augmented datasets for one target subject. The image on the left shows a cross-section of the target subject (Subject1) with the manual segmentations for that image shown in green. The 10 images on the right are cross-sections of the augmented datasets, generated when keeping subject 1 as the target for the registration, whilst using the other 10 subjects as the reference dataset. Segmentations are reported in blue. One augmented dataset marked with a red square did not pass the inclusion criteria, due to the discontinuity in the boundary of the body.
