# Supplementary material for: Deformable image registration based on single or multi-atlas methods for automatic muscle segmentation and the generation of augmented imaging datasets
Source: PLoS One. 2023 Mar 10;18(3):e0273446. doi: 10.1371/journal.pone.0273446 (PMC10004495; doi:10.1371/journal.pone.0273446)
Supplement: S4 File — Comparisons of muscle volumes within the original (11 subjects) and the augmented (69 virtual subjects) databases. The mean volume ± standard deviation and volume ranges are reported. (PDF) [file pone.0273446.s004.pdf]

Body segment	Muscle	Volume (mm^3)			
		Original		Augmented	
		Mean ± Standard deviation	Range	Mean ± Standard deviation	Range
Hips	Adductor brevis	61435 ± 9543	[38576 , 74521]	76561 ± 16900	[39752 , 128029]
	Adductor longus	78821 ± 15755	[59674 , 117231]	67936 ± 15750	[47353 , 137215]
	Adductor magnus	363352 ± 63510	[281549 , 498354]	281756 ± 73115	[178475 , 747305]
	Gluteus maximus	594903 ± 116434	[405632 , 786182]	551872 ± 106214	[311775 , 800670]
	Iliacus	116639 ± 19690	[81848 , 164614]	102010 ± 20623	[65260 , 186217]
Thigh	Tensor fasciae latae	42830 ± 11933	[17359 , 57855]	35486 ± 11185	[9880 , 65367]
	Biceps femoris caput brevis	59201 ± 16047	[31466 , 80666]	53870 ± 18003	[20685 , 85672]
	Biceps femoris caput longum	112310 ± 19158	[78166 , 140450]	103714 ± 22836	[62714 , 186630]
	Gracilis	49664 ± 11848	[34188 , 76124]	38718 ± 9820	[18353 , 62163]
	Rectus femoris	119625 ± 23837	[68701 , 156801]	103714 ± 30949	[39935 , 182029]
	Sartorius	90837 ± 30848	[50854 , 165669]	72742 ± 27104	[30154 , 171743]
	Semimembranosus	122550 ± 20927	[97682 , 154476]	109856 ± 22707	[68140 , 178482]
	Semitendinosus	104117 ± 23925	[63099 , 142124]	89316 ± 20411	[40280 , 157999]
	Vastus intermedius	282828 ± 41849	[213619 , 352606]	241347 ± 43449	[152970 , 375563]
	Vastus lateralis	334628 ± 65168	[245103 , 499165]	296931 ± 67821	[165178 , 441699]
	Vastus medialis	224012 ± 31982	[167408 , 276617]	197803 ± 37837	[135392 , 299946]
Calf	Gastrocnemius lateralis	83069 ± 18458	[47936 , 114767]	63262 ± 14638	[33912 , 101303]
	Gastrocnemius medialis	169092 ± 29193	[122901 , 225733]	143891 ± 33330	[79094 , 234383]
	Peroneus brevis	35953 ± 5288	[24597 , 41577]	28894 ± 9142	[13416 , 59509]
	Peroneus longus	37819 ± 9598	[25693 , 58957]	30866 ± 8476	[16563 , 55113]
	Soleus	340211 ± 69284	[257673 , 502612]	304304 ± 67428	[173345 , 493459]
	Tibialis anterior	95095 ± 17517	[74447 , 135527]	80160 ± 17516	[42534 , 128871]
	Tibialis posterior	77872 ± 15297	[56327 , 109101]	65416 ± 14761	[34707 , 109803]

Table 1: Comparisons of muscle volumes within the original (11 subjects) and the augmented (69 virtual subjects) databases. The mean volume ± standard deviation and volume ranges are reported.
