# Supplementary material for: Deformable image registration based on single or multi-atlas methods for automatic muscle segmentation and the generation of augmented imaging datasets
Source: PLoS One. 2023 Mar 10;18(3):e0273446. doi: 10.1371/journal.pone.0273446 (PMC10004495; doi:10.1371/journal.pone.0273446)
Supplement: S5 File — Statistical analysis and qualitative interpretation of the effects of the pre-processing stage included in the segmentation algorithm. (PDF) [file pone.0273446.s005.pdf]

		Fixed subject				
		1	2	3	4	5
Moving subject	1					
	2	1				
	3		2			
	4					3
	5					

Table 1: Statistical analysis of the effect of homogenising the fat surrounding the muscle tissue on the Dice Similarity Coefficient (DSC) of the segmentation, resulting from registration. A Wilcoxon signed rank test was performed on the segmentation results with and without the pre-processing step. Green squares represent a statistically significant increase in the segmentation accuracy through the pre-processing step of each combination of fixed and moving subject. Additionally, all mean DSCs across the muscles segmented with the pre-processing step were equal to (to 2 decimal places) or greater than those without the pre-processing step. The numbered squares highlight the fixed and moving subject combinations shown in figure 1.

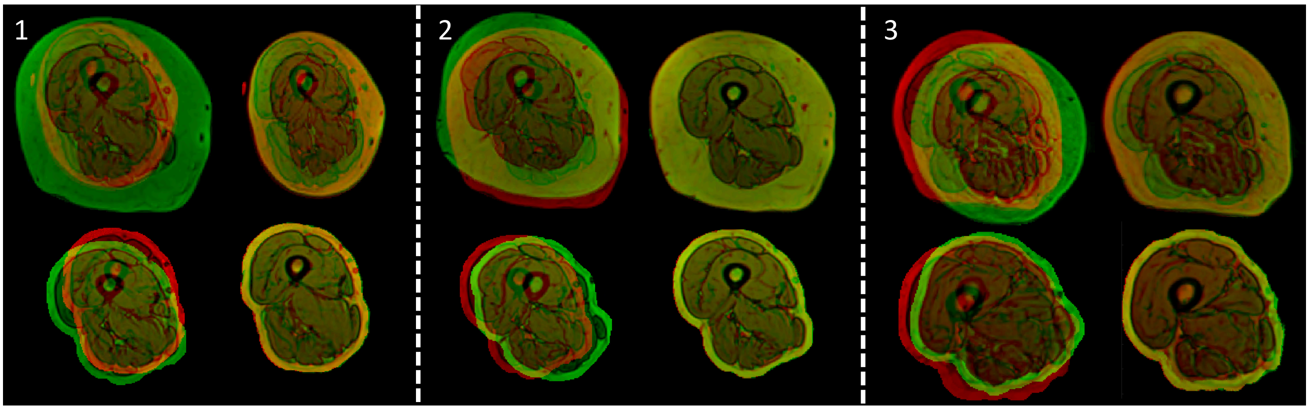

Figure 1: Registration inputs (left column of images in each block) and outputs (right column of images in each block), both with (bottom row of images in each block) and without (top row of images in each block) the pre-processing step. The subject combinations are numbered and highlighted in table 1.
